# Mechanical Dentistry

**Published:** 1871-04

**Authors:** C. M. Wright


### MECHANICAL DENTISTRY.

BY PROF. C. M. WRIGHT.

Read before the Mississippi Valley Dental Association.

*Mr. President and Gentlemen:*

I have not selected a subject chosen by the executive committee, but I beg leave to submit this little paper for your consideration, which will not occupy much of your time. I

want to enter a protest against the neglect of what we call mechanical dentistry, by good men in the profession, and against the extensive use of block teeth in constructing artificial dentures. The arrangement of teeth in Philadelphia in blocks of three, or two, for the convenience of dentists, is a very good thing for making specimen sets of teeth for county fairs, expositions, etc. It is a help to a mechanic, but it is a death-blow to art and artistic or expressional dentistry. The popularity of rubber and cast metal plates with the profession, created the demand for still greater facilities for rapid and cheap dental work, and the block teeth were presented and are now used to the almost total exclusion of single and plain teeth. What is the result? The sensibilities of the people and the profession have been blunted. The artistic aspirations that once filled our hearts have been smothered, and the mechanical dentist is to-day only a mechanic and not at all an artist. He takes an impression of the mouth presented, and without regard to temperament, expression or features, asks of his customer, "Do you like large or small teeth; dark or light teeth?" He then enters his shop, sets up his blocks in a mechanical way, with mechanically good "joints" and mechanical precision, about the alveolar ridge of his model, vulcanizes or casts, polishes, and his work is done.

No wonder "mechanical dentistry" is sneared at and disliked by the more cultivated members of our profession. No wondor that dental colleges are hoping for and working for the abolition of the chair of mechanical dentistry. No wonder a prominent member of this Association, the President of our National Association, publicly expressed in this lecture room a year ago the hope that the day would come when there would be no professors of mechanical dentistry in our colleges. With rubber, the cast metals, and block teeth, what cultivated gentleman can be content to confine himself to the narrow duties of the mechanical business of to-day?

What gentleman of education and refinement and delicate sensibilities can consent to devote his time and strength to disfiguring his fellow men by putting blocks of porcelain in rows around their mouths? We lay loud claims to advancement in the ART OF DENTISTRY in the last twenty or thirty years. We have advanced in the manufacture of teeth and appliances. We can construct, no doubt, with less labor, finer specimens of workmanship. Mechanically we have progressed, but how sadly has *art* suffered? Is not the dental art worthy of our best efforts? Is it not as fine an art to restore or improve expression in the human face as it is to portray this face on canvas? I think it is. Now let us notice our great inconsistency. The operative department, so called, is pointed at with pride by our best men. They are content to spend their lives in the routine of restoring lost portions of teeth with gold.

There is no *art* (in the higher sense of the word) in this. This operation of taking a broken down tooth and building it up and restoring its shape by welding small pieces or pellets, or strips of No. 2 or No. 120, gold foil, is a very delicate, a very nice, a very skillful operation. It is perhaps as delicate a mechanical operation as is known; but it is strictly, purely and only a *mechanical operation*. I refer to the operation itself; of the daily and hourly business of the dental operator. I do not detract from the intelligence and learning necessary to decide upon when the condition of health of the patient and the teeth and to determine what to do. This knowledge should belong to the mechanical as well as the operative dentist? What I wish to assert, and what you will all acknowledge, if you will consider without reference to post sign-boards, labels, and misapplied names, is that the operative dentist is the mechanic in our profession, and that the mechanical dentist, he who mounts and inserts artificial teeth, who selects and arranges porcelain teeth in the mouth, who restores lost expressions of the face, is the *artist*, or has the artistic part of dentistry.

The fact that we save a natural tooth by stopping it with gold, and that we restore its usefulness by building it up to its natural proportions, makes us benefactors. We help men and women and children to be healthier, to breathe better, to eat better, to speak better, by our skill as fillers of teeth. The boot-maker who fits our feet with comfortable, broad soled and neat boots is a benefactor. He helps men and women and children to be healthier, to walk better, to develop their muscles better, to digest their food better. Yet the boot maker is only a skilled mechanic; he is not an artist. The man who rightly performs what we call mechanical dentistry is a benefactor. He helps men and women and children to be healthier when they have been unfortunate, and he also beautifies them; he restores the expressions. He deals with love, hope, fear, joy, anxiety, remorse, affection, and all expressible sentiments. He is an artist. If this is true, should we not, then, discourage the disposition to cast opprobrium on this department? Let us quit glorifying the operative or purely mechanical at the expense of the artistic, and let us banish rubber, cast metals and block teeth, and devote more of our inventive talent and our artistic taste to the artistic part.

We should insist that our students from this time forth shall be taught to manipulate easily the precious metals and to arrange single teeth with reference to expression, etc. Our mechanical professors should be men of taste, of artistic abilities, and the work of our hands should not be judged by the excellency of polish and finish, but by the effect on expression. There are certain rules for producing certain expressions. The actor learns them. He with paint produces mirth or ferocity, intelligence or idiocy, on his own face. The painter knows them. The sculptor knows them. The dentist should also learn them. His best studies are the human face in all its variety, and works of art in marble and on canvas of modern and ancient masters.

Good men should go back to their laboratories, and discarding block teeth, even with the materials we now know, they may restore this almost lost art. Have we the right to devote ourselves to simply filling teeth and leave the art part of our good and respectable profession to boys and men of no taste? Are we wise in trying to discard this part and in clinging to the less troublesome and purely mechanical part, and in condemning mechanical dentistry, because a few leaders who can not aspire to *art* and are only neat mechanics, have chosen to snub this part and shirk it, because the people have so little taste and so poor an appreciation of our best efforts?

These same people have been educated to pay from five to one hundred dollars for an operation on a natural tooth. With the same good service from us they may be educated to an equal appreciation of artistic ability in the miscalled mechanical department.

